# Genomic and Non-Genomic Mechanisms of Action of Thyroid Hormones and Their Catabolite 3,5-Diiodo-L-Thyronine in Mammals

**DOI:** 10.3390/ijms21114140

**Published:** 2020-06-10

**Authors:** Marco Giammanco, Carlo Maria Di Liegro, Gabriella Schiera, Italia Di Liegro

**Affiliations:** 1 Department of Surgical, Oncological and Oral Sciences (Discipline Chirurgiche, Oncologiche e Stomatologiche), University of Palermo, 90127 Palermo, Italy; marco.giammanco@unipa.it; 2 Department of Biological, Chemical and Pharmaceutical Sciences and Technologies (Dipartimento di Scienze e Tecnologie Biologiche, Chimiche e Farmaceutiche (STEBICEF)), University of Palermo, 90128 Palermo, Italy; carlomaria.diliegro@unipa.it (C.M.D.L.); gabriella.schiera@unipa.it (G.S.); 3 Department of Biomedicine, Neurosciences and Advanced Diagnostics (Dipartimento di Biomedicina, Neuroscienze e Diagnostica avanzata (Bi.N.D.)), University of Palermo, 90127 Palermo, Italy

**Keywords:** thyroid hormone metabolism and transport, thyroid hormone mechanisms of action, resistance to thyroid hormones (RTH), 3,5-diiodo-L-thyronine, hepatic steatosis, nonalcoholic fatty liver disease, obesity

## Abstract

Since the realization that the cellular homologs of a gene found in the retrovirus that contributes to erythroblastosis in birds (v-erbA), i.e. the proto-oncogene c-erbA encodes the nuclear receptors for thyroid hormones (THs), most of the interest for THs focalized on their ability to control gene transcription. It was found, indeed, that, by regulating gene expression in many tissues, these hormones could mediate critical events both in development and in adult organisms. Among their effects, much attention was given to their ability to increase energy expenditure, and they were early proposed as anti-obesity drugs. However, their clinical use has been strongly challenged by the concomitant onset of toxic effects, especially on the heart. Notably, it has been clearly demonstrated that, besides their direct action on transcription (genomic effects), THs also have non-genomic effects, mediated by cell membrane and/or mitochondrial binding sites, and sometimes triggered by their endogenous catabolites. Among these latter molecules, 3,5-diiodo-L-thyronine (3,5-T2) has been attracting increasing interest because some of its metabolic effects are similar to those induced by T3, but it seems to be safer. The main target of 3,5-T2 appears to be the mitochondria, and it has been hypothesized that, by acting mainly on mitochondrial function and oxidative stress, 3,5-T2 might prevent and revert tissue damages and hepatic steatosis induced by a hyper-lipid diet, while concomitantly reducing the circulating levels of low density lipoproteins (LDL) and triglycerides. Besides a summary concerning general metabolism of THs, as well as their genomic and non-genomic effects, herein we will discuss resistance to THs and the possible mechanisms of action of 3,5-T2, also in relation to its possible clinical use as a drug.

## 1. Introduction

Thyroid produces two main hormones: L-thyroxine (T4), and L-triiodothyronine (T3). The first one is the predominant form (more than 80%) secreted by the gland and circulating, while T3 is considered the most active form, since it binds with much higher affinity to the nuclear receptors [1,2,3,4,5,6]. In the periphery, the two hormones undergo deiodination, giving rise to other thyronines, some of which have be found to have hormonal activity [7,8,9].

Thyroid hormones (THs) are among the regulatory factors with the highest number of effects in the human body. They do so through different mechanisms, the best understood of which rely on their ability to bind nuclear receptors [1,10,11,12,13,14]. As discussed below, in the absence of active hormone, the receptors associate with co-repressors and inhibit chromatin transcription, while, upon binding with THs, they release co-repressors, bind co-activators, and stimulate transcription of those genes they had inhibited before [15]. The real situation is actually more complex because some nuclear receptor-mediated TH effects do not involve a direct binding of receptors to DNA [16]. In addition, some rapid TH effects are mediated by hormone binding to plasma membrane sites, such as α_v_β_3_ integrin [17], or to other cytoplasmic sites [14,18]. Finally, some catabolic products, once considered inactive, such as 3,5-diodothyronine (3,5-T2), have been more recently found to have important effects in the organism [19,20].

Obesity is an important risk factor for cardiovascular, degenerative, and malignant diseases [21,22,23,24,25]. Overeating can cause mitochondrial dysfunction, mainly in white adipose tissue (WAT). Mitochondrial function alteration may in turn result in an altered substrate oxidation and increased oxidative stress [26]. These events foster development of obesity and associated pathologies [27,28,29,30,31]. It is now clear that THs regulate the expression of several genes involved in lipolysis, lipogenesis, thermogenesis, mitochondrial function, and nutrient availability [32]. Given their action on metabolism, it seemed, in the past, that they could be used as pharmacological agents for the treatment of obesity. However, this approach could be not applied, due to adverse side effects on many organs and systems and, in particular, on the cardiovascular system and the heart’s rhythm [33,34]. Yet, in recent years, it has been found that some metabolites of thyroid hormones, and especially 3,5-diiodo-L-thyronine (3,5-T2), are endowed with interesting metabolic activities that may be of clinical interest as possible therapeutic options in the treatment of overeating disorders [20].

Herein, we will summarize some central aspects of TH metabolism and cellular action, both at the genomic and non-genomic level. In addition, we will discuss resistance to THs and the possible mechanisms of action of 3,5-T2, also in relation to its possible clinical use for the treatment of lipid dysmetabolism and obesity.

## 2. Thyroid Hormone (TH) Metabolism

Synthesis and release of thyroid hormones is strictly controlled by the hypothalamic–pituitary–thyroid axis (HPT axis) [35,36,37]. In response to a variety of physiologic and environmental stimuli, hypothalamic neurons of the paraventricular nucleus (PVN) secrete the thyrotropin-releasing hormone (TRH), which stimulates the anterior pituitary to produce the thyroid-stimulating hormone (TSH). TSH regulates, in turn, all the steps of thyroid growth and function since the late fetal life [38]. On the other hand, production of TRH and TSH is subjected to a negative control by THs [39]. HPT functioning thus ensures constant levels of THs in the circulation. The epithelial cells of the thyroid gland (thyrocytes) have the ability to concentrate iodide ions, thanks to a specific sodium/iodide symporter (NIS or SLC5) present in their basolateral membrane [40,41,42], the synthesis of which is stimulated by TSH [42]. In the case of iodine shortage, hypothyroidism follows. From this point of view, it is of note that fluoride is able to inhibit NIS functioning through different molecular mechanisms, such as inhibition of the Na^+^/K^+^-dependent ATPase, and upregulation of cytokines (including interleukins IL-6 and IL-1β, and TGFα), that in turn inhibit biosynthesis of NIS [43]. 

Once in the epithelial cell, iodine is translocated across its apical membrane (iodide efflux) through other transporters [42]. One of these transporters seems to be pendrin; however, there is no general agreement about its actual role [19].

### 2.1. TH Synthesis, Release, and Transport to the Target Cells

**Figure 1:**
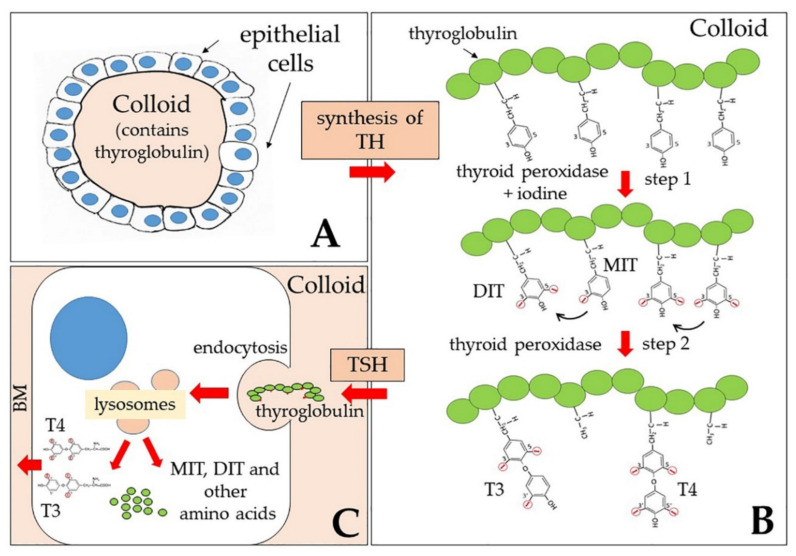
Schematic drawing of the reactions leading to T4 (thyroxine) and T3 (triiodothyronine) production and release from the thyroid gland. (**A**) General structure of one of the thyroid follicles: Each follicle is formed by a layer of epithelial cells lining a central space filled with colloid, mainly formed by the thyroglobulin; (**B**) synthesis of thyroid hormones (THs) starts by addition of iodine atoms to position 3 or to both 3 and 5 positions of the aromatic ring of various tyrosine residues of thyroglobulin (step 1). One mono-iodinated (MIT) or a di-iodinated (DIT) ring of a tyrosine residue is then transferred (black arrows) to the ring of an adjacent di-iodinated tyrosine, thus forming triodo-thyronine or tetraiodo-thyronine residues, respectively (step 2). The positions 3 and 5 of the outer ring are now indicated as 3′ and 5′. Both step 1 and step 2 are catalyzed by a thyroid peroxidase and take place in the lumen of follicles. (**C**) From time to time, as a response to thyroid stimulating hormone (TSH), endocytosis of thyroglobulin into the epithelial cells that line the follicle and fusion of endosomes with lysosomes allow digestion of thyroglobulin by proteases and release of the hormones T3 and T4, as well as of other amino acids, including MIT and DIT. While these latter remain in the gland, T3 and T4 are released into the blood through the basal membrane (BM).

The main products of the thyroid gland are L-thyroxine (T4) and triiodo-thyronine (T3). Their synthesis involves different enzymatic steps, the first of which is iodination at position 3 or at both positions 3 and 5 of the aromatic ring of L-tyrosine residues, present in the protein thyroglobulin (TG) (Figure 1). The mono-iodinated (MIT) or di-iodinated (DIT) ring of a tyrosine residue is then transferred to the ring of another di-iodinated tyrosine, thus forming triodo-thyronine or tetraiodo-thyronine residues, respectively, both of which contain an inner tyrosine ring and an outer phenolic ring; these residues are the immediate precursors of T3 and T4. Both synthetic steps are catalyzed by a thyroid peroxidase (TPO), and take place in the lumen of thyroid follicles, in the so-called colloid, mainly formed by thyroglobulin [42]. Both TG and TPO are thyroid differentiation markers and function as autoantigens in thyroid autoimmune diseases like the Hashimoto thyroiditis [42]. 

From time to time, as a response to the thyroid-stimulating hormone (TSH), endocytosis (both macro- and micropinocytosis) of thyroglobulin from the apical surface of thyrocytes that line the follicle and fusion of endosomes with lysosomes allow digestion of thyroglobulin by proteases and release of the hormones, as well as of other amino acids, including MIT and DIT (Figure 1). Thyroglobulin thus functions both as a precursor for TH synthesis and as a storage of inactive hormones in the lumen of follicles. 

Once secreted, THs are bound to TH distributor proteins (THDPs) that transport them in the circulation. There are two main TH carriers in the blood: The thyroxine-binding globulin (TBG), a monomeric protein of 54 kDa that shows higher affinity for THs, and transthyretin (TTR, previously called thyroxine-binding prealbumin, TBPA), a symmetrical tetramer of 55 kDa, rich in beta sheet structures [44,45]. In addition, albumin also binds THs even if with lower affinity than TBG and TTR. Carriers have a dual function: They control the amount of free hormone, exchangeable with the cells [1,37], and are responsible for the uniform distribution of circulating THs throughout the body [44,45]. In addition to the above-mentioned carriers, it has been reported that THs and some of their metabolites can also bind to low-density lipoproteins (LDL) [46].

Free THs enter the cells through transmembrane transporter proteins and not, as it had been believed in the past, by passively crossing the plasma membranes. Since the 1970s it was found, indeed, that TH transport was a saturable process. In more recent years, a variety of carriers have been found to be involved in TH transport across the plasma membrane. Among them, three families play a major and more specific role, as indicated by the effects of their mutation: (1) The monocarboxylate transporters (MCT) 8 and 10, (2) the L-type amino acid transporters (LATs), and (3) the organic anion transporters (OATPs, SLC10, and SLC17) [37,47,48,49]. MCT8 is also present in the basolateral membrane of thyrocytes and is critical for secretion of THs [37,47]. Mutations of MCT8 were already reported to be associated with severe intellectual disability many years ago [50] and, more recently, it was found that mutations in the OATP1C1 transporter associate with severe brain hypometabolism and juvenile neurodegeneration [49,51]. These observations demonstrated that serious symptoms of hypothyroidism can be caused by deficits of TH membrane transporters, and the resulting inability of the hormones to enter the cells in sufficient amount.

### 2.2. Activation and Inactivation of THs by Deiodinases

**Figure 2:**
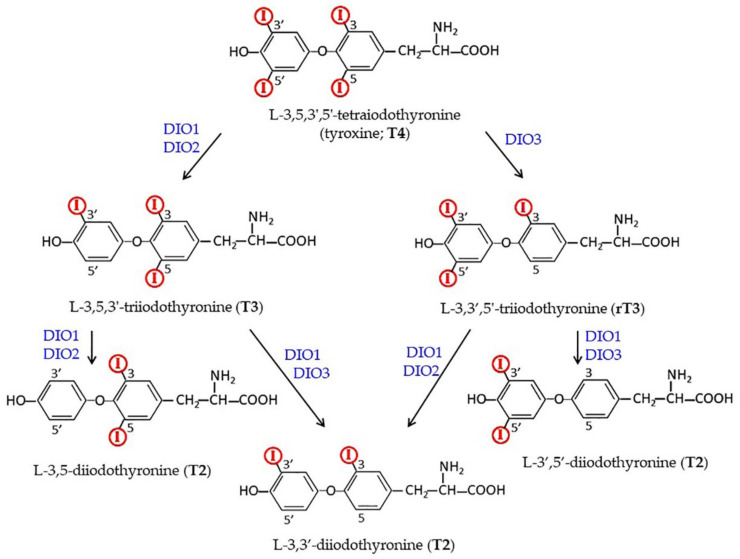
Metabolic pathways leading from the prohormone T4 to the active hormone T3 and to other inactive/active derivatives of THs (see below). Deiodinases probably involved in each transition are indicated.

As mentioned, the main form of THs secreted from the thyroid gland and circulating is T4, while T3 is considered the active hormone as it binds with the highest affinity to the nuclear receptors [1,2,3,4,5,6]. Although a small amount of T4 is converted to T3 in the thyroid itself, conversion of T4 into its active form takes place mainly in the target cells (Figure 2). Both in the thyroid and in the periphery, metabolism of THs involves deiodinases (DIOs), a family of enzymes that consists of three members: (1) DIO1 that catalyzes deiodination of both the outer and inner rings of iodothyronines, (2) DIO2 that catalyzes deiodination of the outer, phenolic ring (ORD), and (3) DIO3 that catalyzes deiodination of the inner tyrosine ring (IRD) [37]. All DIOs belong to the thioredoxin fold superfamily and contain a selenocysteine in the active site [8,48,52]. The three isoforms differ for intracellular localization: DIO1 and DIO3 localize to the plasma membrane, while DIO2 is an endoplasmic reticulum resident protein. Moreover, under ischemia/hypoxia, DIO3 is rapidly redirected to the nuclear envelope, where it inactivates T3 [3]. DIO2 has been demonstrated to play a critical role in the brain, where it is mainly present in astrocytes: T4 enters these latter cells, is deiodinated to T3, and is then transferred through MCT8 to neurons, where it binds to TH nuclear receptors or is degraded to 3,3′-T2 by DIO3 [3,53]. Notably, DIO3 is encoded by an imprinted gene, and its expression changes during development as well as across different regions of the brain. The epigenetic nature of DIO3 expression regulation introduces a further level of temporal and regional modulation of brain cell ability to respond to THs [54].

DIO2 has been also reported to play a central role in T4-mediated feedback mechanisms in the HPT axis: In the hypothalamus, DIO2 is highly expressed in tanycytes, specialized ependymal cells located close to the TRH-producing neurons of the PVN. Tanycytes receive T4 and transform it into T3, which then reaches PVN and the pituitary gland [3,55]. A special property of DIO2 is its relatively short half-life, due to TH-dependent, post-translational regulation by ubiquitination and degradation by the proteasome [56]. Interestingly, it has been reported that the polymorphism Thr92-to-Ala (Ala92-DIO2) causes endoplasmic reticulum (ER) tress, thus activating the unfolded protein response (UPR). Ala92-DIO2 tends, indeed, to accumulate in the trans-Golgi. As a consequence, less T3 is produced; this situation can be improved by eliminating ER stress with the chemical chaperone 4-phenyl butyric acid (4-PBA) [56]. Notably, Ala92-DIO2 polymorphism has been also associated with insulin resistance [57]. In particular, it has been suggested that a decreased local production of T3 because of DIO2 malfunctioning can influence the expression of the insulin-sensitive glucose transporter 4 (GLUT4), in adipocytes and skeletal muscle cells; the gene encoding GLUT4 is indeed a T3 target gene [58].

The three classes of deiodinases also differ for their tissue distribution: Under physiological conditions, DIO1 is mainly expressed in the thyroid gland, liver, and kidney; DIO2 in the thyroid gland, brain, skeletal muscle, and brown adipose tissue; and DIO3 in brain and pancreas [8,59]. 

As shown in Figure 2, both DIO1 and DIO2 are able to transform T4 into T3, with DIO2 having a higher affinity for the hormone (Michaelis-Menten constant, K_m,_, in the range of 1-4 nm) than DIO1 (K_m_ in the range of 1–0 μm) [8]. Given the already mentioned differences in the half-life and localization of the two enzymes, however, it is not easy to establish which enzyme has the highest impact on local T3 formation. Intriguingly, it has been reported that the DIO1/DIO2 double knock-out (KO) mice appear healthy, and even show normal serum T3 levels [60]. The authors suggested that the two enzymes were not essential for the maintenance of the serum T3 level, but that they (and in particular DIO2) did have an important role in thyroid hormone local homeostasis [9,60].

## 3. Cellular Mechanisms of Action of THs

The best known mechanisms of action of THs rely, similarly to steroids, on direct effects on transcription regulation, mediated by nuclear receptors. These effects become evident after a time lag during which synthesis of RNA and proteins is required. However, some cellular activities are immediately stimulated by THs, suggesting that other mechanisms are also in action. As reported below, these further activities are collectively indicated as “non-genomic”.

### 3.1. Thyroid Hormone Receptors (TRs)

**Figure 3:**
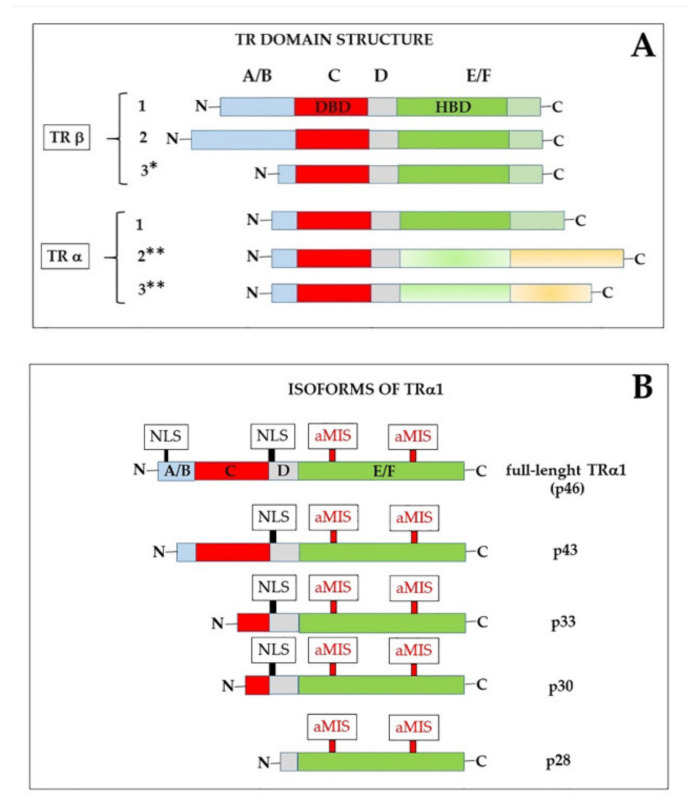
(**A**): Schematic drawing of the domain structure of thyroid hormone nuclear receptors (TRs). Each isoform contains: (1) An N-terminal A/B domain, of variable length, (2) a highly conserved DNA-binding domain (DBD, or C domain), (3) a hinge D domain, (4) the hormone-binding domain (HBD or E domain), and, finally, (5) a C-terminal domain (F domain). * This isoform was only found in rat [77,89]. ** These isoforms are not able to bind hormones [12,77]. (**B**) The mature mRNA encoding TRα1 can give rise to different isoforms, depending on the AUG from which translation begins. The truncated isoforms are shorter at the N-end, but have an unaltered hormone-binding domain. NLS, nuclear localization signal; MIS, atypical mitochondrial import sequences (MIS), that mediate import of p43 and p28 to the mitochondrial matrix and the inner mitochondrial membrane, respectively [14,18] (discussed in Section 3.3).

The existence of receptors for THs (TRs) in the nucleus was already known since the 1970s, and it was also shown that they had a much higher affinity for T3 than for T4 [9,61,62]. In the 1980s, when different steroid receptors, as well as the TRs, were cloned, it became evident that they all belonged to a superfamily of structurally related nuclear proteins that recognized specific DNA response elements present in the target genes [63,64,65,66]. In particular, TRs were identified as the products of the cellular c-erbA α and β proto-oncogenes, present on human chromosomes 17 and 3, respectively [65,66,67]. The two genes were demonstrated to give rise, by alternative splicing, to different variants, some of which were not able to bind THs because the splicing events eliminated part of the hormone-binding domain [68,69,70,71,72]. Notably, one of these splicing isoforms not able to bind T3 (α2) is highly expressed in the brain [12,73,74]. As shown in Figure 3A, α and β isoforms share a common structure [10,11,12,75,76,77,78], that includes: (1) An N-terminal A/B domain, of variable length, endowed with modulatory functions and an effect on transcription transactivation (activation function 1, AF1). The A/B domain also contains a nuclear localization signal (NLS) fundamental for TRα1 import into the nucleus (see below). The activity of this domain can be modified by post-translational events, such as phosphorylation [79] and acetylation [80]; (2) a highly conserved DNA-binding domain (DBD, C domain), which includes two “zinc fingers”, able to interact with the DNA double helix. In particular, in each zing finger, four invariable cysteines form a tetrahedrical coordination structure with a Zn^2+^ ion. Some amino acids present at the base of the first Zn-finger are required for recognizing specific DNA motifs [81], while some residues of the second zinc finger are involved in receptor dimerization [75]; (3) a hinge domain (D) that contains a NLS for both TRα1 and TRβ and can participate in interactions with receptor co-regulators. It has been also proposed that the D domain might form extensions of the DBD and the hormone binding domain (HBD) or unfold to permit TRs to adapt to different thyroid hormone response elements (TREs) [82]; (4) HBD (E domain), that contains residues necessary for hormone recognition as well as multiple contact surfaces that mediate homo- and hetero-dimerization and the interaction with other regulatory proteins; and, finally, (5) a C-terminal domain (F domain), endowed, like the N-terminal domain, with transactivation of transcription function (AF2) [67]. 

Much of the action of TRs depends on interaction with the already mentioned DNA sequences called TREs, present in the regulatory regions of the TH target genes and formed by two half-sites, each including at least the AGGTCA hexamer consensus motif, separated on average by four nucleotides [12,16,72,78,83]. The two half-sites can be organized as direct repeats as well as palindromic and inverted repeats [78,81,84]. DNA motifs recognized by different nuclear receptors can differ for the sequence of the two half-sites as well as for the number of nucleotides present in between them. For example, it was shown that TREs containing direct repeats can be converted into retinoic acid response elements (RREs) by increasing the spacing between the half-sites by one single nucleotide, while, decreasing the spacing, again by a single nucleotide, converted them into vitamin D3 response elements (DREs) (81). These early results were a clear demonstration of the importance of the spacing between the repeats for determining the affinity and specificity of DNA motifs for different regulatory molecules. Notably, the hexamer sequence of the two half-sites can also differ in different natural TREs, suggesting that variability of the repeats might influence responsiveness of different genes to THs [85]. 

In most cases, nuclear TRs form heterodimers with the retinoic acid X receptors (RXR) that also belong to the superfamily of nuclear receptors. In particular, the RXR component of the heterodimer was found to bind to the 5′ repeat of the TRE, while the TR protein binds to the 3′ repeat [84]. However, in some cases, TRs bind to DNA as monomers or homodimers [86], and it was demonstrated that each species (TR, TR/TR, and RXR/TR) exhibits preferences for different TREs [87,88]. Some isoforms of TR β have been even reported to bind as trimers to some naturally occurring TREs [83].

**Figure 4:**
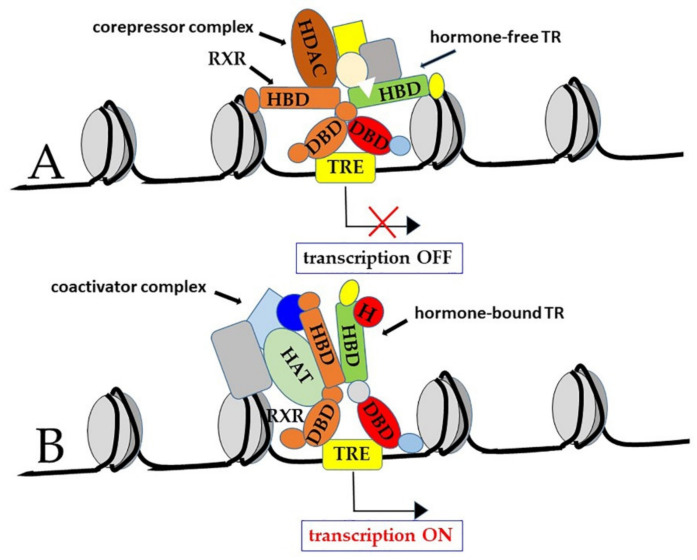
Canonical model of TR interaction with thyroid hormone responsive elements (TRE). When TRs are bound to ‘positive’ TREs in the hormone-free form, they are part of co-repressor complexes that repress transcription (**A**). On the other hand, in the hormone (H)-bound form, TRs form co-activator complexes that activate transcription (**B**). Although TRs can bind to chromatin as monomers as well as homodimers, they often bind TREs as heterodimers with the retinoic acid X receptor (RXR), another member of the nuclear receptor family; in this latter case, the RXR component of the heterodimer was found to bind to the 5′ repeat of the TRE, while the TR protein bound to the 3′ repeat.

Another classic paradigm of TR function states that most of the known TREs are ‘positive’ regulators at which transcription is repressed by TRs, in the absence of hormone, and activated by T3-bound TRs. However, some TREs are ‘negative’ regulators that operate in the opposite direction: Transcription is stimulated by hormone-free TRs, while it is repressed by T3-bound TRs [11,76,85,90,91]. Both positive and negative effects of TRs depend on interaction with co-regulators, known as “co-activators” and “co-repressors”, respectively [37,92,93,94] (Figure 4). 

In more detail, when TRs are bound to ‘positive’ TREs in the hormone-free form, they are part of co-repressor complexes that include the nuclear corepressor (NCoR), the silencing mediator for RXR and TR (SMRT), and histone deacetylases (HDAC). These latter enzymes also associate with methyl-CpG-binding proteins, thus contributing to methylation-dependent gene silencing [12,37,95].

On the other hand, in the hormone-bound form, TRs are part of coactivator complexes that include the steroid receptor coactivator 1 (SRC-1), also known as nuclear coactivator 1 (NCoA-1), the transcriptional intermediary factor 2 (TIF2/GRIP-1/NCoA-2), the cAMP-response element binding protein (CREB)-binding protein (CBP), also known as p300, the p300/CBP-associated factor (p/CAF), the vitamin D receptor-interacting protein/TR-associated protein (DRIP/TRAP), and the mediator complex subunit 21 (MED/21, also known as Srb7) [75,96,97,98,99,100]. Notably, many co-activators have histone acetyltransferase (HAT) activity [101,102,103]. Histone acetylation is indeed a well-known mechanism to remodel chromatin structural organization in order to allow interaction of the basal transcription factors with the gene promoters [104]. In some cases, HAT can also acetylate non-histone proteins, including basal transcription factors themselves, such as TFIIE and TFIIF [105]. Probably, in many cases, T3-bound TRs can function as pioneer factors, able to “open” chromatin structure, allowing other transcriptional factors to gain access to gene promoters [16,106].

As mentioned above, in a few cases, hormone-bound TRs repress transcription. Although the mechanisms underlying this second type of hormone action are less clear, it seems that TRs might interfere, in a T3-dependent manner, with the activity of other transcriptional factors, thus inhibiting their function and, as a consequence, gene transcription. For example, it has been reported that both TRβ1 and TRα1 interfere with the binding of the TEAD/TEF transcription factors (TEADs) to a specific sequence present in the promoter of the gene encoding myosin heavy chain 7 (MYH7), in a T3 dose-dependent way. As a consequence, TEADs that are the main activators of MYH7 gene transcription in the heart of rodent embryos are no longer able to activate transcription after birth, when circulating T3 concentration increases [107]. Similarly, T3-bound TRs are able to inhibit the expression of the gene encoding the β subunit of TSH (TSHβ) [108,109,110,111] and the gene encoding DIO2 [112], in the pituitary, by a tethering mechanism that prevents the binding of pituitary-specific transcription factors, such as Pit-1 and GATA2 [112].

**Figure 5:**
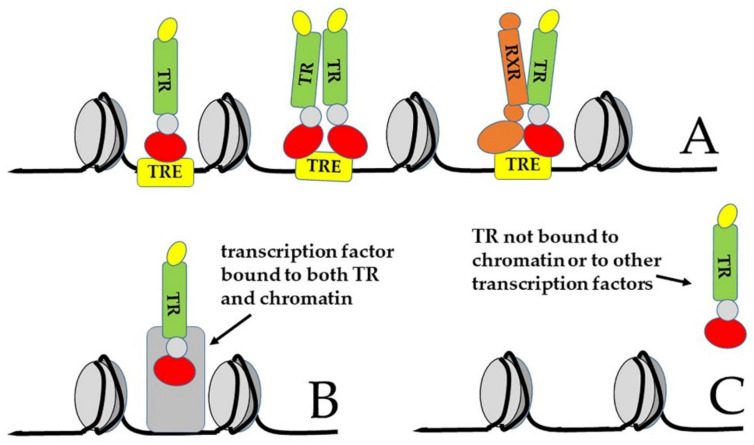
Possible alternative ways of action of TRs. (**A**) Canonical way of action: TRs can interact with DNA elements called thyroid hormone responsive elements (TRE) as monomers, as homodimers, or as heterodimers with RXR; (**B**) TRs can interact with chromatin in an indirect way, by tethering to another chromatin-bound protein; (**C**) the action of TR can be independent of both direct and indirect chromatin binding [16].

More recently, indeed, it became clear that the canonical model drawn in Figure 4 and Figure 5A is not the only mechanism of action of TRs [14,16,72,113]. For example, experiments based on chromatin immunoprecipitation and sequencing (ChIP-Seq) demonstrated that TRs can be bound to sequences other than TREs, sometimes not present in the gene promoters [88,114]. Thus, it has been envisaged that TRs can also interact with chromatin in an indirect manner, by tethering to other chromatin proteins (Figure 5B, type 2 of TR action). In addition, their activity can be even independent of both direct and indirect chromatin binding (Figure 5C, type 3 of TR action); in this latter case, TR can function both in the nucleus and in the cytoplasm [16]. Finally, as we will discuss in Section 3.3, TH action can be completely independent of the nuclear receptors (type 4 action of thyroid hormones) [16], although at least some of these so-called “non-genomic” functions of TH actually rely on truncated isoforms of TRs that localize to parts of the cell other than the nucleus [14,15,18,115].

On the basis of the complexity of TR mechanisms of action, it can be expected that a further level of regulation of TR action is their trafficking between nucleus and cytoplasm. Recently, it has been found that nuclear entry of TRα1 is mediated by interactions of the nuclear transport proteins importin 7, importin β1, and the adapter importin α1 with the nuclear localization motifs (NLS1 and NLS2) present on the receptor [116]. Interestingly, TRβ1, which lacks NLS-2, tends to show a slightly more cytosolic localization [14,80]. Similarly, exit from the nucleus requires nucleus export signals (NES) that are present in the hormone-binding domain [117]. As a whole, these observations suggest that the nuclear action of THs also depends on the amount of active TRs that enter the nucleus [118]. As a consequence, TR post-translational modifications, by tuning this ability, can also affect TH activity. For example, TR phosphorylation modulates DNA binding, gene transcription, and even nuclear localization [79]. Moreover, both the DBD and HBD have been demonstrated to undergo sumoylation [119]. Interestingly, it has been recently reported that non-acetylated forms tend to be retained in the nucleus, while, on the contrary, acetylation promotes cytosolic localization [80]. A further example of post-translational modification is ubiquitination that targets hormone-bound TRs for degradation [120].

As we will discuss in the next paragraph, the described functions of TRs can be affected at many levels; for example, their genes can harbor mutations that inhibit, or completely abolish, their ability to bind DNA or hormone; alternatively, epigenetic modifications of chromatin organization of TR encoding genes can inhibit or enhance TR production. These events, together with the already mentioned genetic or epigenetic modifications of transporters and enzymes involved in TH metabolism and delivery to tissues, can cause apparent conditions of hypo- or hyperthyroidism, in the presence of normal concentrations of circulating hormones. 

### 3.2. TH Resistance 

Notably, the three hormone-binding TR isoforms have different tissue distributions [71,72,121]: TRα1 is predominantly expressed in bone, central nervous system, cartilage, heart, skeletal muscle, and gastrointestinal tract; TRβ1 in liver and kidney; and TRβ2 in hypothalamus and pituitary (where it is responsible for the feedback control exerted by THs on the HPT axis) [122], as well as in inner ear and retina. Although the affinity for T3 is quite similar for all the isoforms, as mentioned above, each kind of TR can have a different intracellular localization pattern. Most important, the unequal α and β isoform tissue distribution brings about different effects on the organism in case of mutation of the corresponding gene (THRA or THRB, respectively). 

The existence of peculiar syndromes due to resistance to thyroid hormones (RTH) has been known for more than five decades [123]. In more recent years, the cause of these syndromes was attributed to mutations of THRA or TRHB, and the corresponding syndromes are now called RTHα and RTHβ, respectively [37,72,123,124,125]. As can be expected from the main mechanism of action of TRs, mutations affecting the HBD (E/F domain) interfere with hormone binding to TR and have a dominant negative effect on THs action: TRs remain stably bound to the TREs in the repressive hormone-free form [77,121,126,127,128]. On the other hand, mutations that involve the DBD are much less serious than mutations that involve the HBD: When TRs are not able to bind DNA, target genes are probably left in neutral state, in which they are neither activated by hormone-bound TRs nor repressed by hormone-free-TRs and, indeed, the dominant negative effect of a TRβ, mutated in the HBD, can be counteracted by introducing into the DBD a mutation that impedes binding to TREs [129]. Moreover, it has been found that mutations of the co-repressor NCoR that abolish its interaction with TR [130], or treatment with inhibitors of HDAC [131], in a murine model of RTHα, both improve phenotypic abnormalities. Recently, it has been reported, however, that treatment of mice with the HDAC inhibitor suberoylanilide hydroxamic acid (SAHA) does not seem to improve the skeletal disorders due to RTHα [132].

**Figure 6:**
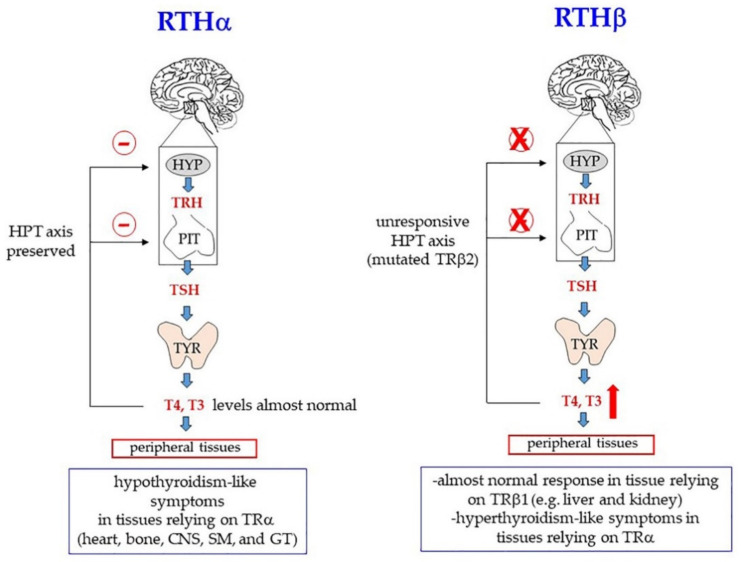
Mutations in genes encoding the thyroid hormone receptor α (TRα, gene: TRA) and the thyroid hormone receptor β (TRβ, gene: TRB) cause resistance to thyroid hormones (RTH), with different effects on peripheral tissues, depending on the TR isoform expressed. In the presence of inactivating mutations of TRα (RTHα), tissues relying on this isoform of TR experience a hypothyroidism-like condition: the hypothalamic-pituitary-thyroid (HPT) axis is indeed unaffected. Thus, THs exerts a normal negative feedback (the red minus sign, in the figure), and there is no compensatory increase of circulating THs. In the presence of inactivating mutations of TRβ (RTHβ), mutated TRβ2 does not bind T3 and does not inhibit production of TRH and TSH in the hypothalamus or pituitary, respectively; thus, T4/T3 levels increase. As a consequence, peripheral tissues relying on TRβ1 isoform (liver and kidney) can have almost normal responses to THs because the increased levels of circulating THs compensate for the decreased ability of TRβ to bind them. On the other hand, tissues relying on TRα experience a hyperthyroidism-like condition because of the increased levels of THs. CNS, central nervous system; SM, skeletal muscle; GT, gastrointestinal tract.

The first case described of a familial RTH syndrome was characterized by deaf-mutism, stippled epiphyses, goiter, and abnormally high levels of protein-bound iodine [123]. Since then, many other clinical symptoms have been identified that can be attributed to target organ resistance to thyroid hormones. Based on analyses done in Europe, North and South America, and Asia, the incidence of resistance (about 90% RTHβ) is estimated to be around 1:50,000 live births [72]. Notably, given the different tissue distribution of TRα and TRβ, the pathological outcomes are different, depending on the mutated TR gene. The most common signs of RTHβ are goiter, attention deficit disorders, and resting tachycardia, but severity of the symptoms can vary depending on the specific mutation in TRB gene and even among individuals who bear the same mutation [72]. Probably, the main defects underlying RTHβ correlate with TRβ2-dependent modulation of the HPT axis: When TRβ is mutated, the negative feedback, normally exerted by THs on the hypothalamus and pituitary, is impaired; as a result, the levels of TSH tend to increase and the thyroid gland is continuously stimulated, so that both its volume (goiter) and TH production increase. TH increase partially compensates for TRβ resistance in those tissues that rely on TRβ. On the other hand, tissues that rely on TRα (for example, the heart) experience a hyperthyroidism-like condition (Figure 6, RTHβ) [37,72,133]. 

Up to now, more than 160 mutations in TRβ (mostly single nucleotide substitutions, but also deletions, frameshifting, and nonsense modifications that generate stop codons) have been identified. Most TR mutations are familial; some, however, are sporadic [72,134,135]. 

Mutations in THRA are rare and account only for a small proportion of all TR genetic modifications. For decades no THRA mutation was identified at all, and it was even believed that TRα loss of function might have been incompatible with life. To date, about 20 missense and frameshift mutations of THRA have been described [121,136,137,138,139,140]. Clinical features of patients include many symptoms commonly present in hypothyroidism, such as bradycardia, constipation, skeletal dysplasia (macrocephaly, late fontanelle closure, and epiphyseal dysgenesis), delayed bone growth and psychomotor development, and intellectual disability [72,121,141]. In other words, the pathological signs involve those tissue that normally rely on TRα. On the other hand, the HPT axis is unaffected and there is no compensatory increase of circulating THs (Figure 6, RTHα). As in the case of RTHβ, a large variability of severity can be observed, depending on the specific mutation [137]. 

In one patient affected by all the symptoms described above, also epilepsy was observed [142]. Moreover, a de novo missense mutation (R384C) in TRα1 was found in a patient affected by an autism spectrum disorder [143]. Recently, it has been reported that TRα1 is required for normal neural progenitor cell proliferation in human cerebral cortical development; as a consequence, mutations in the TRA gene cause a reduction of brain size and intellectual disability [141].

Notably, somatic mutations in TRs have been also found in some cancers, such as human hepatocellular carcinoma, renal clear cell carcinoma, breast cancer, pituitary tumor, and thyroid cancer [14,144,145]. These observations suggested that TRs can also have a tumor suppressive role [146,147].

As a final comment on TH resistance, it is worth noting that, in some patients with RTH, no mutation was found in TRs [148], in their co-regulators [149], or in enzymes involved in TH transport and metabolism. The same observation has been done for cancer cells. Now, a variety of recent studies suggest that deregulation of gene expression is very often caused by epigenetic factors, such as alteration of DNA methylation pattern and/or of histone post-translational modifications. Moreover, it is becoming increasingly clear that epigenetic effects can also depend on deregulation of the expression of specific microRNAs (miRNAs), short RNAs, about 22 nucleotides in length, that recognize complementary sequences (microRNA recognition elements, MREs) present on target mRNAs: Pairing results in the repression of target mRNA translation or even in its degradation [150]. In line with this idea, it has been reported, for example, that in renal cancer, miR-155 and miR-425 are upregulated and that their increase is inversely correlated with the cell content of TRβ [151]. Similarly, studies performed in cardiac and skeletal muscle have suggested that a group of miRNAs (including miR-133, miR-208a, miR-208b, and miR-499) might mediate/modulate TH signaling in these tissues [152,153].

### 3.3. Non-Genomic TH Action

In addition to the mechanisms of action already discussed, some TH functions can be, at least in part, also independent of nuclear receptors (type 4 of thyroid hormone action) and take place outside the nucleus [16]. The extra-nuclear pathways are defined “non-genomic” [15,154,155,156,157,158,159] to distinguish them from the canonical regulation of gene transcription, defined “genomic”, and mediated by TRs. However, they can eventually affect transcription, too, and can be mediated by alternatively spliced isoforms of TRs (see below). 

More than five decades ago, the existence of TH receptors at the plasma membrane had been already hypothesized, and these sites were proposed to be involved in the rapid onset of some cellular responses to the hormones, even elicited in conditions of transcriptional blockage [1,160,161,162]. A large body of evidence then confirmed that THs can trigger signal transduction pathways initiated at the plasma membrane and often mediated by secondary messengers, such as Ca^2+^ ions, inositol trisphosphate (IP3), and cAMP [154,155,163,164]. Moreover, a plasma membrane-binding site was identified as integrin αvβ3, a member of the family of proteins that mediate the bidirectional interaction between the cell and the extracellular matrix (ECM) and regulate the organization of tissues as well as cell migration processes [15,157,165,166]. The αvβ3-dependent pathway is mostly sensitive to T4 and activates intracellular kinases, such as protein kinase B (PKB/AKT), and the mitogen-activated protein kinase (MAPK) that in turn phosphorylate intracellular proteins, some of which act in the nucleus and can regulate transcription. Notably, this pathway can also stimulate cell proliferation and may have a role in different cancer types, such as T-cell lymphoma (TCL), colorectal cancer (CRC), and glioma [166,167,168]. By binding to αvβ3 in cancer cells, T4 activates transcription of a variety of genes that can stimulate cancer growth, such as genes with a function in signal transduction, angiogenesis, regulation of actin cytoskeleton, and epithelial-mesenchymal transition [168,169,170]. Interestingly, non-malignant cells express less αvβ3 than normal cells and, in addition, the conformation of the protein in normal cells seems to have a lower signal transduction activity [168]. 

The hormone-binding site on αvβ3 was initially identified with the Arg-Gly-Asp (RGD) amino acid sequence that also mediates cell binding to ECM proteins [171]. More recently, however, it has been suggested that the receptor site is more complex and includes a T3-binding site (S1) besides a T3/T4 binding site (S2), with a lower affinity for T3. Since, in physiological conditions, T3 is much less concentrated than T4 outside the cell, the probability that T4, and not T3, binds S2 is much higher [166,172]. The signaling triggered by T3 binding to S1 involves the phosphoinositide 3-kinase (PI3K)/PKB(AKT) pathway, which does not seem to regulate cell proliferation, but can modulate the shuttling of regulatory proteins, including TRα, from the cytoplasm to the nucleus [15,172].

In fact, activation of PI3K was also suggested to be mediated by a direct binding of cytoplasmic TRβ to the regulatory subunit (p85) of the enzyme [173]. The ability to bind the regulatory subunit of PI3K has been also reported for TRα1 [174]. In any case, activation of PI3K stimulates transcription of genes, such as the hypoxia-inducible factor (HIF)-1α [173]. 

The plasma membrane receptor site present on integrin αvβ3 is not the only extra-nuclear binding site for THs. Other “receptors” are also present in the cytoplasm. Notably, four proteins (p43, p33, p30, and p28) have been identified, which are encoded in the same mature mRNA that also encodes TRα1 [175]. This mRNA can be also translated starting at internal AUG triplets, thus resulting in proteins that are shorter than TRα1 at the N-end [14] (Figure 3B). The full-length protein contains, as mentioned, at least two signals for nuclear localization (NLS); in addition, it contains atypical mitochondrial import signals [176] that probably are not active when both NLS are present. The truncated translation products lack one or both NLS and their localization might thus be influenced by other signals. We can also suppose that exposure of the localization signals is influenced by the conformation of the proteins, and that conformation can be influenced by the length of the N-terminus. 

In human primary osteoblasts (hPOBs) and mouse osteoblast-like MC3T3 cells, the TRα1 truncated isoform p30 was found to be modified by palmitoylation and inserted into the inner leaflet of the plasma membrane, where it colocalized with caveolin-1 [115]. In this position, p30 could bind THs, thus activating a signal transduction pathway that resulted in increased intracellular concentrations of calcium ions, nitric oxide (NO), and cyclic guanosine monophosphate (cGMP), leading to activation of the cGMP-dependent protein kinase II (PKGII) [115]. On the other hand, in rat liver mitochondria prepared by differential centrifugation and purified through sucrose gradients, p43 was found to localize to the mitochondrial matrix [177]. Finally, p28, the activity of which still remains unclear, was found at the level of the mitochondrial inner membrane [18]. Gel shift experiments demonstrated that p43 can also recognize canonical TREs, with an affinity similar to that of nuclear full-length receptors. Notably, the mitochondrial genome contains four TRE-like sequences that can bind p43 in gel shift experiments [18]. Moreover, it was found that over-expression of p43 induced large increments of both mitochondrial activity and mitochondriogenesis; these effects can be abolished by deleting the DNA-binding domain of the protein [18]. 

Interestingly, it was also reported that there is mitochondrial localization of TRα2, the dominant negative isoform lacking a functional HBD. Moreover, by electromobility shift assays, it was shown that TRα2 binds to TREs present in the 12S rRNA gene and D-loop region of mitochondrial DNA [178].

All these findings, together with the fact that mitochondrial isoforms of other hormone receptors, including mtRXR, have been also identified [179], provide evidence that thyroid hormones have direct effects on mitochondria and, probably, with mechanisms that, at least in part, resemble the nuclear ones. An interesting hypothesis is that a TH-mediated interplay may exist between the nuclear and the mitochondrial genome [180]. The framework becomes even more complex when we consider the existence of TH metabolites with specific roles, only partially superimposable to those of the main THs. In the next paragraphs we will consider, in particular, the hormonal properties of 3,5-T2.

## 4. The 3,5-Diodothyronine (3,5-T2) as a Hormone

In spite of their wide effects on metabolism, the use of THs for therapeutic purposes remains controversial because, as mentioned, these hormones may induce other non-metabolic effects. In more detail, like hyperthyroidism, treatment with exogenous THs can induce thyrotoxicosis, a condition characterized by a variety of adverse symptoms, such weight loss, osteoporosis, atrial fibrillation, embolic events, and increased risk of heart failure [33,34,181]. 

In the last two decades, however, much attention has been devoted to TH metabolites, and especially to 3,5-T2. As discussed below, indeed, 3,5-T2 has some T3-like effects [182,183,184,185,186,187] in the absence of thyrotoxic side effects, at least when used at low concentrations [20,188]. On the contrary, at higher concentrations, it has been reported to suppress TSH and the HPT axis [189,190,191] and even to cause adverse cardiac effects similar to those observed in hyperthyroidism [190].

Early studies indicating a physiological role of 3,5-T2 were initially overlooked because the molecule was considered only an inactive metabolite of thyroid hormones T3 and T4. This interpretation was based on the observations that 3,5-T2 had a low affinity for TRs [113,183]. Thus, most of the available studies on THs mainly dealt with T3; as discussed above, indeed, thyroid hormone receptors showed a higher affinity for this TH form. However, at the end of 1980s and the first years of the 1990s, after finding that thyroid hormones also had non-genomic effects, the putative role of 3,5-T2 was re-evaluated and investigations began to be directed at assessing the metabolic effects of this molecule [183,192,193,194,195,196].

As summarized below, many experimental observations have shown that 3,5-T2 administration to rodents caused an increase in the resting metabolic rate (RMR) [185,197] and prevented overweight and insulin resistance induced by a high-fat diet (HFD) [198,199]. Furthermore, the administration of 3,5-T2 to HFD rats seems to protect animals from the onset of non-alcoholic fatty liver disease (NAFLD) [191], although this finding has been challenged by results obtained in Sprague–Dawley rats fed an unsaturated fat diet, in which 3,5-T2 failed to improve NAFLD and insulin sensitivity [200]. It is worth noting, however, that 3,5-T2 has been suggested to influence glucose metabolism not through effects on insulin sensitivity, but by reducing hepatic glucose transporters 2 (GLUT2) and glucose output from the liver [201].

In humans, demonstration of 3,5-T2 physiological effects and, in particular, of its benefits on obesity and related diseases is still equivocal because of the lower number of experiments performed, and also for the difficulties encountered in measuring 3,5-T2 concentration in human serum. An antibody-based competitive chemiluminescence immunoassay (CLIA) [202] was used to investigate serum concentration of 3,5-T2 in humans under both physiological and pathophysiological conditions; the results suggested that 3,5-T2 concentrations do not differ in hyperthyroid (0.31 ± 0.02 nm) compared to hypothyroid (0.43 ± 0.04 nm) individuals [202]. More recently, HPLC coupled to tandem mass spectrometry [203] allowed a more accurate 3,5-T2 detection in human serum (the average concentration reported was 78 ± 9 picomoles/liter) [203]. In general, significant differences still remain among different detection methods. Thus, the data reported in the literature are not uniform. In addition, given the already discussed existence of different DIOs, with different activities, in peripheral tissues, we cannot exclude that the concentration of 3,5-T2 is different in tissues with respect to blood. A further problem arises from the degree of purity of commercial preparations of 3,5-T2, used as standards in the measurements; these preparations are, indeed, frequently contaminated with T3 [20,204]. Even with these limitations, 3,5-T2 is now widely considered a bioactive molecule with dose-dependent effects on physiological regulatory functions [20,59,113,191,204,205,206,207,208,209,210,211]. 

### 4.1. The 3,5-T2 Production in the Cell and Its Effects on Rest Metabolic Rate (RMR)

Many observations suggested that 3,5-T2 might derive from T3 by deiodination (Figure 2). As an example, both T3 and 3,5-T2 stimulated oxygen consumption by isolated perfused livers from hypothyroid rats at a concentration as low as 1 pM within 90 min, but 3,5-T2 gave rise to a faster stimulation than T3 [212]. Moreover, inhibition of DIOs by propylthiouracil (PTU) abolished the rapid stimulation of oxygen consumption by T3, but not the effects of 3,5-T2. These data suggest that the peripheral deiodination of T3 is a key metabolic step in the production of 3,5-T2 and that this latter molecule has the same power as T3, but exerts its effect more rapidly because it does not require deiodination [212]. Starting from these observations, further studies were carried out to better understand the effects of 3,5-T2 on energy metabolism, and on cellular respiration. The administration of 3,5-T2 to experimental animals was confirmed to induce more rapid effects, on oxidative phosphorylation (OXPHOS), than those obtained with T3: While 3,5-T2 activity was evident one hour after the injection, T3 activity was evident only after 24 h [213,214]. Notably, the effect of 3,5-T2 was found to be independent of protein synthesis: It was not influenced by the protein synthesis inhibitor cycloheximide [215]. These early experiments also suggested that the effects of 3,5-T2 were mediated by a direct effect on mitochondria, while those of T3 depended primarily on nuclear events [194].

It follows that, if 3,5-T2 can influence the rate of energy consumption by mitochondria, the hormone probably has an effect on the energy metabolism of the whole animal. This hypothesis was also supported by previous studies in which the changes of the RMR, after administration to hypothyroid rats of single doses of 3,5-T2 or T3, had been monitored and compared [216,217,218]. From these experiments, however, it was not clear whether the effects on RMR were effectively due to the hormones or to their metabolites. This is the reason why, in other experiments, as mentioned, deiodinases were simultaneously inhibited by the administration of propylthiouracil (PTU) and iopanoic acid (IOP). This treatment induced a marked inhibition of all the three types of deiodinases, and, at the same time, severe hypothyroidism in animals. In particular, PTU inhibited the production of thyroid hormones by the thyroid gland, via inhibition of both the thyroid peroxidase activity, and DIO1. On the other hand, IOP had no influence on the production of thyroid hormones, but exerted an inhibitory effect on all the forms of deiodinases, including DIO2 and DIO3 [219]. Similar investigations showed that, in hypothyroid animals, in which RMR was significantly reduced compared to euthyroid animals, both T3 and 3,5-T2 were able to significantly increase RMR. The administration of a single dose of T3 in rats, resulted, indeed, in approximately a 35% RMR increase, which occurred 25–30 h after T3 injection, and rose to a peak after 50–75 h. The T3-induced effect lasted up to 5–6 days after administration. On the other hand, the same dose of 3,5-T2 induced a 40% RMR increase after 6–12 h, which rose to a peak at 24–30 h and lasted 48 h [193]. Notably, if injected into euthyroid rats, T3 had both early and late effects, but when injected together with actinomycin D, a drug able to block transcription, the late effects of T3 were no more evident, while the early effects were still present, thus confirming the idea that some T3 effects might be due to its intracellular deiodination to 3,5-T2 [185,193]. As a whole, these findings supported the hypothesis of a mechanism of action of 3,5-T2 that ruled out the transcriptional processes and which was independent of the nucleus. In other words, 3,5-T2 might be involved in short-term effects induced by THs in physiological situations in which an increase in energy expenditure was required. An example is the exposure to cold, a situation in which additional energy is required to counteract the increase in heat loss. THs play a key role in cold adaptation processes, as indicated by the experiments showing that hypothyroid rats survive to cold temperature only for 3–4 days [220]. There is strong evidence that, although with different mechanisms, both 3,5-T2 and T3 may increase the resistance to cold [220]. In cold-exposed hypothyroid rats, 3,5-T2 and T3 increase animal energy expenditure and stimulate the oxidative capacity expressed in terms of Cytochrome C Oxidase (COX) activity of tissues with a high metabolic rate, such as heart, skeletal muscle, liver, and brown adipose tissue (BAT) [220]. However, while the stimulating activity of T3 on the trophic activity of tissues are probably due only to its nuclear effects, the specific targets of 3,5-T2 appear to be mitochondria, thus resulting in an improvement of the oxidative capacity of tissues. 

In some experiments, in order to assess the specific effects induced by 3,5-T2 and T3 and to rule out those of their metabolites, hypothyroidism was induced in rats by the concomitant administration of PTU and IOP. Then, the daily energy expenditure was determined by continuous monitoring of oxygen consumption and CO_2_ production. In hypothyroid rats, in which daily energy expenditure was low, the administration of 3,5-T2 and T3 restored the normal values observed in euthyroid rats. The evidence that 3,5-T2 is a metabolically active hormone was provided, in the same study, by the finding that it also had stimulating effects on β oxidation of fatty acids [192]. All the 3,5-T2 effects reported above were observed in experiments carried out in rats with a deficit of thyroid function. In order to investigate the effects of 3,5-T2 in normal conditions, single injections of T3 were given to euthyroid rats and the results were compared with those obtained after T3 administration to animals rendered hypothyroid and treated, at the same time, with the above-mentioned deiodinase inhibitors [185]. The results of these studies showed that: (1) The effect of T3 on RMR of euthyroid rats occurred approximately 25 h before those observed in rats treated with PTU + IOP; (2) the first phase of the change in RMR of euthyroid rats treated with T3 appeared to be very similar to that observed following 3,5-T2 administration; (3) the administration of T3 and actinomycin D to rats made hypothyroid by PTU + IOP treatments caused a marked reduction of the early effects of the hormone, thus highlighting the need of deiodination to produce the early effects of T3; and (4) the highest increase in the rate of RMR concurred with the highest hepatic concentration of 3,5-T2. Overall, these results confirmed that the early increase in the rate of RMR, occurring after the administration of T3 in euthyroid rats, was likely to be due to its conversion to 3,5-T2 by deiodinases, an effect which is independent of actinomycin D [185]. 

All these findings indicated that the precursor of 3,5-T2 in vivo is T3. However, many attempts to demonstrate a direct deiodination of T3 to give 3,5-T2 failed [204]. These findings, as well as the high degree of 3,5-T2 variability in serum, hamper the complete understanding of the mechanisms that generate 3,5-T2. Thus a few other hypotheses have been proposed: (1) In spite of all the above reported evidences, T3 might not be the precursor to 3,5-T2; (2) for still-unknown reasons, T4/T3 concentration in the blood might be not directly related to serum and/or tissue levels of 3,5-T2; (3) transformation of T3 to 3,5-T2 might happen with different kinetics in different cell types and might depend on tissue-specific factors; and, finally, (4) 3,5-T2 might be rapidly metabolized to other bioactive molecules.

### 4.2. Cellular Targets of 3,5-T2

Several observations support the hypothesis that mitochondria are the specific target of 3,5-T2. For example, after an in vitro pre-incubation of liver homogenate with radioactive 3,5-T2, specific binding sites, with high affinity (10^-8^ moles/liter) and low binding capacity (0.4–0.6 pmoles/mg protein) were detected in rat liver mitochondria [221]. Competition experiments provided evidence that unlabeled 3,3′-T2, T3, and T4 could all compete with 3,5-T2 for the same sites, but only when added at high concentrations [221]. Notably, after isolation of mitochondria, a significant increase in the activity of Cytochrome C Oxidase (COX) could be evidenced [222,223]. The effect was proposed to be mediated by a direct binding of 3,5-T2 to COX that induced a conformational change of the oxidized enzyme, evidenced by spectral changes [223]. This hypothesis was in agreement with previous analyses that had identified two sites of the mitochondrial respiratory chain as targets of 3,5-T2, namely complex IV (i.e., COX), which transfers electrons from the reduced cytochrome c to O_2_ to produce H_2_O, and the reductases involved in the reduction of cytochrome C [224]. More specifically, the Va subunit of the COX complex was identified as a critical binding site for 3,5-T2 [225]. The same study also showed that the effects of 3,5-T2 on the COX complex consisted of a suppression of the allosteric inhibition of COX by ATP, as the Va subunit is adjacent to the IV subunit, which binds ATP [225]. These findings also stimulated a search for cytosolic 3,5-T2 binding sites, possibly involved in transferring the hormone to the mitochondria. By photo-affinity labeling of rat liver cells, three proteins able to bind 3,5-T2 were identified, with apparent molecular masses of 86, 66, and 38 kD, respectively [226]. In particular, the 38 kD protein was able to bind either 3,5-T2 or T3, but its affinity for 3,5-T2 was more elevated, and 3,5-T2 binding was independent of NADPH concentration. Conversely, binding of the 38 kD protein to T3 was NADPH-dependent. Thus, this cytoplasmic factor, by acting in a cellular redox state-dependent manner (i.e., depending on the NADPH/NADP ratio), seemed to function as a reservoir of 3,5-T2 and T3, and as a carrier as well [226]. A further effect of 3,5-T2 on mitochondria was suggested to depend on increased absorption of mitochondrial Ca^+2^ [227]. Importantly, it has been known for a long time that Ca^+2^ ions might trigger an enhancement of mitochondrial activity, due to the increased activity of at least three mitochondrial dehydrogenases (i.e., pyruvate dehydrogenase, isocitrate dehydrogenase, and α-ketoglutarate dehydrogenase), which could increase the amount of reduced substrates available for the respiratory chain [228]. 

The effects of 3,5-T2 are not limited to mitochondria. In myocytes of newborn rats, T4, T3, and 3,5-T2 were indeed found to significantly increase Na^+^ currents, as compared to untreated controls. In contrast, as also observed for reverse T3 (rT3), MITs or tyrosine, the acidic metabolites of T3 and T4 (tetraiodothyroacetic acid, TETRAC, and triiodothyroacetic acid, TRIAC, respectively) had no effect on Na^+^ currents. These acute effects of THs and their analogues on Na^+^ currents were attributed to non-genomic mechanisms of action. T3 and 3,5-T2 also affected membrane transport systems, such as the Na+/H+ exchanger and the amino acid transport system of chicken embryo hepatocytes [229]. Evidence was also provided on rapid non-genomic effects of THs during fetal development and cellular differentiation: 3,5-T2 was able, in these studies, to mimic some of the effects of T3, but less efficiently [230]. Thus, it is possible that 3,5-T2 is also involved in some of the non-genomic T4/T3 effects described in Section 3.3. On the other hand, long-term effects of 3,5-T2 on de novo lipogenesis (see Section 4.3) have been also reported [231]. Interestingly, 3,5-T2, at a concentration of 0.1–1.0 μm, can also modulate energy metabolism in cardiomyoblasts, in both ex vivo and in vitro models; in particular, this effect was found to be based on an increase of glucose consumption [232], as also shown in skeletal muscle [233].

### 4.3. The 3,5-T2 and Lipid Metabolism

NAFLD is a pathological condition, widespread in wealthy societies, and characterized by altered lipid metabolism, with consequent accumulation of fat in hepatocytes, increased oxidative stress, and abnormal production of cytokines [234,235,236]. NAFLD is associated with visceral obesity and cardiovascular risk factors [24]. This condition can also evolve into steatohepatitis, advanced fibrosis, cirrhosis, and finally hepatocellular carcinoma [237]. The ectopic development of fat storage in the liver was associated with alterations in the mitochondrial compartment. Both rats with steatotic liver and patients with steatohepatitis showed a reduced OXPHOS capacity, peripheral insulin resistance, and increased oxidative stress in hepatic mitochondria, although the ability to use fat as a metabolic fuel was increased [29,238,239]. The increase in fatty acid oxidation stimulates, in turn, ketogenesis as a compensatory mechanism. Increased mitochondrial β-oxidation has been also observed in the liver of genetically obese (ob/ob) mice, with massive steatosis [240]. However, the increase in lipid oxidation is not sufficient to handle the increased burden of hepatic free fatty acids (FFAs), so the remaining FFAs are converted into triglycerides, which are partly deposited in the cytoplasm, thus causing steatosis.

Although the modulation of the caloric intake and increase of physical activity are the cornerstones of the treatment of metabolic disorders [241,242,243], some experimental and clinical investigations were aimed at finding out novel drugs that could be useful for the prevention and/or treatment of steatosis. Thus, a variety of TRβ-specific agonists have been prepared and shown to cause significant decrease of cholesterolemia and body weight [244,245,246,247,248]. For example, TRC150094, a functional analogue of iodothyronine has been shown to reduce adiposity in HFD rats [249]. 

Besides using TH analogues and TR agonist, the effects of 3,5-T2 were evaluated in rats fed a HFD over a long period. The results obtained in these studies indicated that in HFD rats, 3,5-T2: (1) Reduced body weight and metabolic efficiency, without suppressing TSH levels; (2) decreased the serum levels of cholesterol and triacylglycerols (TAG); (3) increased the hepatic mitochondrial consumption of O_2_ and oxidation of fatty acids; (4) activated the proton mitochondrial dispersion in hepatocytes; and (5) reduced the mitochondrial oxidative stress [250]. An interesting aspect concerns the changes in efficiency of substrate utilization in steatotic animals. The decrease in proton loss and the increase in the body weight/caloric intake ratio clearly indicate a more efficient use of energy in these animals in comparison with healthy controls. On the other hand, after 3,5-T2 treatment, the efficiency of the energy use clearly decreased. While T3 action on mitochondria is mainly mediated by nuclear induction of the synthesis of uncoupling proteins (UCPs), fundamental for OXPHOS uncoupling, and thermogenesis [251,252,253,254,255,256], as discussed in the previous paragraph, 3,5-T2 effect is probably mediated by a direct interaction with the COX respiratory complex [223,225]. Interestingly, in hypothyroid rats treated with 3,5-T2, besides the already mentioned increase of oxygen consumption, a rise in the ATP synthase activity could be observed; this effect could be explained, at least in part, by increased levels of cardiolipin [257]. Notably, however, an increase of both mRNA and proteins levels of the enzyme subunits was also observed, thus suggesting a T2-induced nuclear effect [258]. In general, 3,5-T2, contrary to T3, does not induce proton leak (i.e., it does not induce synthesis of UCPs), but is somehow able to influence the kinetic properties of the respiratory pathways [259].

Notably, administration of 3,5-T2 for four weeks induced a further increase in β-oxidation and the carnitine palmitoyltransferase (CPT) system activity. These changes, responsible for a better compensation of the hepatic load of FFAs, could be one of the mechanisms that make 3,5-T2 able to improve steatosis. Notably, the increase in the rate of mitochondrial respiratory processes could enhance the re-oxidation of the reduced coenzyme NADH to NAD+, necessary for both β-oxidation and the tricarboxylic acid cycle. All together these actions would lead to a higher fat consumption [250]. Actually, administration of 3,5-T2 decreases hepatic mitochondrial oxidative stress, as indicated by the significant decrease of H_2_O_2_ [250] (paragraph 4.4).

The role of 3,5-T2 in HFD rats was also evaluated, in comparison with euthyroid rats receiving a standard diet (N) [198]. The results of these experiments showed that in HFD-T2, 3,5-T2 was able to reduce both adiposity and serum levels of free fatty acids, triglycerides, and cholesterol, without inducing any clinical sign of thyrotoxicosis. In HFD animals, β-oxidation levels were 30% higher, the activity of acetyl-coenzymeA-carboxylase (ACC) significantly lower (−65%), and the activity of carnitine palmitoyl-transferase system (CPT) 38% higher than in N animals. Notably, the levels of AMP-activated protein kinase (AMPK), which inhibits ACC activity in different physiological conditions, were also decreased. The reduced ACC activity observed in HFD rats was probably due to a reduced amount of the enzyme, rather than to its decreased activity. Thus, the increase in fatty acid oxidation in HFD rats may be due to a reduced level of malonyl-CoA (the product of the reaction catalyzed by ACC) that, in turn, allows activation of the CPT system (normally inhibited by malonyl-CoA) [198]. Treatment with 3,5-T2 further increased fatty acid oxidation by significantly activating AMPK and CPT activity, without increasing CPT1 mRNA levels and without further inhibiting ACC activity [198]. These results suggest that 3,5-T2 increases fatty acids entering into the mitochondria by regulating CPT1 activity in an AMPK-dependent manner and ACC-malonyl-CoA-independent manner [198]. The possible existence of a malonyl-CoA-independent control mechanism on the hepatic activity of CPT1 is supported by some studies that showed that stimulation of hepatic fatty acid oxidation can also use a malonyl-CoA-independent pathway that involves an AMPK-mediated phosphorylation of cytoskeletal components, leading to CPT1 stimulation [260,261]. Notably, the increase in body weight and adiposity observed in HFD rats clearly indicates that: (1) An increase in hepatic fatty acid oxidation is not sufficient, per se, to prevent fat accumulation, due to HFD continuous consumption, and (2) the TH-dependent reduction of adiposity is due to a less efficient use of the substrates. One well-known TH effect is the ability to cause OXPHOS uncoupling by allowing the energy of the proton gradient to be dissipated as heat [262]. In HFD-T2 rats, 3,5-T2 caused a less efficient use of lipid substrates by reducing the synthesis of ATP and inducing a greater fat combustion, which was indeed more elevated in HFD-T2 than in HFD rats [198]. The physiological consequences of these effects were an increase in energy expenditure and a slight increase of body temperature. This suggested that proton gradient dissipation had a decisive role in the effects exerted by 3,5-T2 on substrate utilization efficiency and, consequently, on adiposity [198]. 

To assess the hypothesis that 3,5-T2 acts on rat hepatocytes through nuclear receptor-independent mechanisms, its effects were also studied in a well differentiated rat hepatoma cell line (FaO) [263], which lacks functional TRs, as also demonstrated by the absence of constitutive mRNA expression for both TRα1 and TRβ1. An in vitro model of “steatosis” was developed [264,265] by exposing FaO cells for 3 h to a mixture of oleate/palmitate (2:1, 0.75 mm final concentration), which closely mimics plasma FFA levels of patients with metabolic syndrome [266]. 

Normally, FFAs that enter the liver undergo esterification to produce TAGs, which are then stored, for later use as metabolic fuel, in the form of cytosolic lipid droplets (LDs). Typically, LDs are composed of a core of neutral lipids surrounded by phospholipids and proteins of the PAT family, among which the adipose differentiation-related protein (ADRP), and the tail-interacting protein of 47 kDa (TIP47) [267,268,269]. Among these proteins, ADRP is involved in adipocyte differentiation and promotes lipid incorporation in LDs, while inhibiting FAA oxidation. Its expression increases in liver steatosis, and in model animals exposed to HFD [269,270], and is under the control of peroxisome proliferator-activated receptors (PPARs) [270,271]. 

PPARs play a key role in lipid metabolism [272,273]. PPARα improves catabolism and lipid mobilization [274], while PPARγ promotes lipid synthesis and LD formation [275,276,277] and PPARδ increases the synthesis of high-density lipoprotein (HDL), inhibits LD formation in the liver, improves FFA catabolism, and promotes energy uncoupling in adipose and muscular tissues [278,279]. Furthermore, PPAR β/δ play a crucial role in hepatic lipid homeostasis and insulin sensitivity, by activating glycolysis and lipogenesis [280]. The relative concentrations of the different PPARs change depending on both physiologic and pathological conditions [281]; moreover, specific lipid mixtures used to feed both the animals and the isolated cells can have a significant impact on the dynamic equilibrium of PPAR regulation [264,282]. All the PPAR subtypes (PPARα, PPARγ, and PPARδ) are constitutively expressed in FaO rat hepatoma cells [275,276]. Notably, treatment of steatotic FaO cells with 3,5-T2 or T3 for 24 h reduced TAG content as well as the number and size of LDs. The effects of 3,5-T2 were also accompanied by a reduction of PPARγ [282,283]. These findings demonstrated that the direct hypolipidizing effect exerted both by 3,5-T2 and T3 on the hepatic cells can occur even in the absence of TRs [264,265,282] and are based on the ability to activate pathways affecting TAG deposits within LDs and to promote mitochondrial oxidation and/or exocytosis of the very low density lipoproteins (VLDL) [284]. The 3,5-T2-mediated effects in steatotic hepatocytes in culture also include the recruitment to LDs of the adipose triglyceride lipase (ATGL), which might be an early mediator of iodothyronine action [282,285]. However, it has been also reported that neither T2 nor T3 increased AMPK-mediated phosphorylation of ATGL, while, on the basis of a metabolomics analysis, lipophagy (i.e., autophagy of lipids) seemed to be the major mechanism at work for triglyceride digestion, at least in the first phase of hepatic adaptation to HFD [286].

Recently, in primary human hepatocytes, freshly prepared from donors and grown on Matrigel, not only 3,5-T2 but also 3,3′-T2 were able to reduce lipid accumulation, by decreasing expression of lipogenic enzymes. Moreover, it was found that they do so by inhibiting the mammalian target of rapamycin complex 1 (mTORC1), through an AMPK-mediated effect, and activating mTORC2 [211]. 

T3 normally also stimulates hepatic fatty acid and cholesterol syntheses. Both syntheses are also fundamental for the turnover of cell membrane components. These T3 effects are mediated by its binding to TRs, and by the consequent transcriptional activation of genes encoding lipogenic enzymes; genes encoding enzymes like ACC and fatty acid synthase (FAS) contain, indeed, TREs in their promoters [287]. Moreover, TREs are also contained in the promoter of the gene encoding the transcription factor known as carbohydrate response element-binding protein (ChREBP), which can exert a further stimulatory effect on transcription of the lipogenic genes [287]. In HFD rats treated with T2 (25 μg/100 g of body weight), expression of all these genes, and activity of the sterol regulatory element-binding proteins 1c (SREBP-1c), also involved in activation of lipogenic genes, is downregulated [288]. On the other hand, in the same rats, T3 (2.5 μg/100 g of body weight) did not repress ChREBP, even if it repressed expression of SREBP-1c (this latter gene contains a negative TRE in its promoter) [288]. Interestingly, in the HEPG2 cancer cells, it was found that 3,5-T2 can block the proteolytic cleavage (and, thus, activation) of SREBP, without any effect on its expression [289]. In the same experiments, it was also found that apoptosis of HEPG2 cells seemed to occur, after 12 h of T2 treatment [289]. 

The effect of 3,5-T2 seems to be the opposite in the liver of hypothyroid rats, in which the hormone, after a long-term treatment (15 μg/100 g of body weight, for 1 week) was found to activate genes involved in de novo lipogenesis, by increasing the amount of the nuclear forms of SREBP and ChREBP [231]. Although further studies are necessary to better understand the mechanisms underlying these effects, it may be hypothesized that the 3,5-T2 nuclear effects observed in hypothyroid rats are due to SREBP and ChREBP, while 3,5-T2 should have an indirect effect, due to its ability to activate a still-unknown pathway leading to SREBP/ChREBP activation.

### 4.4. Effects of T2 on Lipid Peroxidation

A high-fat diet presumably provides more long-chain FFA capable of entering hepatocytes, thus inducing upregulation of PPARα, which, in turn, controls the expression of genes involved in mitochondrial and peroxisomal fatty acid β-oxidation, as well as in microsomal omega-oxidation [290,291]. The expression of PPARα, and of genes regulated by it, increases indeed in the liver of obese rodents fed fat-rich diets [292,293,294]. These effects have been also highlighted in liver biopsies from patients with NAFLD [295]. 

Fatty liver mitochondria oxidize FFA at a higher rate and produce higher amounts of superoxide when compared to mitochondria from normal liver [296,297]. In addition, increased oxidation of long chain FFA in peroxisomes produces higher amounts of hydrogen peroxide (H_2_O_2_), in the reaction catalyzed by acyl-CoA oxidase (AOX) [298]. The excess of reactive oxygen species (ROS) in turn induces a higher level of liver lipid peroxidation [234].

Hepatocytes try to counteract the cellular oxidative stress by increasing the expression of antioxidant enzymes. Fatty liver mitochondria show a 70% greater manganese superoxide dismutase activity [296], and peroxisomal enzymes (i.e., catalase) involved in H_2_O_2_ metabolism are also stimulated in HFD [298]. The cell protective mechanisms activated in HFD also include the synthesis of antioxidant molecules such as glutathione [299], and metallothioneins (MTs). MTs are low-molecular weight (6–7 kDa), highly conserved, and ubiquitous cysteine-rich proteins, with high affinity for divalent metals; they are involved in some essential biological functions, including homeostatic regulation of zinc and copper availability, detoxification from heavy metals, and scavenging of free radicals [300,301,302,303,304]. A number of stimuli induce their expression, including acute phase response, cold and heat stress, and some hormones, such as the growth hormone (GH) [305].

Notably, transgenic mice over-expressing MT are more protected from hepatic oxidative stress associated with alcoholic liver disease [306]. On the other hand, the observation that MT I/II knockout mice become mildly obese, suggests a role for MTs as modulators of energy metabolism [307]. MTs also play a key role in liver damage and liver regeneration [308,309]. Evaluation of the transcription profiles of the two isoforms constitutively expressed in the liver, namely, MT-1 and MT-2 [300], highlighted upregulation of both isoforms in rats fed HFD, thus suggesting their potentially protective role in the liver. In addition, in obese subjects, MT-2 mRNA levels are also increased in the adipose tissue [310]. ROS can induce the expression of MTs through the interaction with the antioxidant response elements (ARE), and with the metal response elements (MRE), present in the MT gene promoters and recognized by different factors, including the metal-responsive transcription factor 1 (MTF-1) [311,312,313]. Finally, it is worth noting that MTs are located in the intermembrane space of the mitochondria of the liver cells and, when imported into liver mitochondria, they can inhibit respiration [314].

All the above-described effects of HFD were partially neutralized by the administration of 3,5-T2 to rats. Treatment with 3,5-T2 reduces the activities of both superoxide dismutase (SOD) and catalase (CAT), thus suggesting that iodothyronines have a protective effect against excessive FFA oxidation [283,298]. After 30 days of 3,5-T2 treatment, upregulation of PPARα was also counteracted. Moreover, 3,5-T2 prevented both lipid peroxidation (measured as thiobarbituric-reactive substances (TBARS)) and the increase of MT-2 in the liver [298]. Interestingly, 3,5-T2 did not alter the transcription profile of MTs in the liver of rats fed a standard diet, thus suggesting that the effects of 3,5-T2 on hepatic expression of MTs are secondary to the effects of the hormone on molecules and/or pathways associated with fat intake. In particular, the effects of 3,5-T2 might be explained by a selective stimulation of the metabolism of fatty acids in the mitochondria, at the expense of peroxisomal and microsomal oxidation. It is also possible that the effects of 3,5-T2 on the liver depend on a reduced influx of fat in hepatocytes, caused by a primary effect of 3,5-T2 on other tissues, such as the adipose tissue. 

Importantly, an increase of oxygen consumption and of oxygen radical formation also cause damage to the mitochondrial DNA (mtDNA), that is, per se, more vulnerable than the nuclear. The 3,5-T2, by reducing mitochondrial oxidative stress, has been found to be able to also reduce mtDNA damage [315]. 

### 4.5. Effects of 3,5-T2 on Adipose Tissue

Two main kinds of adipose tissues, with different ontogenic origin and lineage, exist in the body: The white adipose tissue (WAT) and the brown adipose tissue (BAT) [32]. WAT stores triglycerides, but also produces adipokines [316]. On the other hand, BAT dissipates energy in the form of heat [32].

Endotherm animals have the ability to maintain a quite constant body temperature almost independently of environmental conditions. This ability is fundamental as it is directly related to the animal performance in the everyday life [317]. When heat loss is higher than heat production through basal metabolism, there are two possibilities for producing extra heat: Shivering and non-shivering thermogenesis [318]. Shivering response is given by muscle contraction, while non-shivering thermogenesis depends on nervous signals transmitted from the brain via the sympathetic nervous system (SNS) to BAT. Transmission of these signals allows release of noradrenalin at the level of BAT and activation of triglyceride hydrolysis, followed by free fatty acid β-oxidation in the mitochondria [318]. An important role in this process is played by the uncoupling protein 1 (UCP1); as discussed in Section 4.3, UCP1 allows proton leak across the mitochondrial internal membrane, thus uncoupling OXPHOS and allowing dissipation as heat of part of the energy stored in the proton gradient [319]. OXPHOS, per se, already releases a considerable amount of heat since not all the enthalpy deriving from substrate oxidation is converted into ATP synthesis and this basal heat production is particularly effective in tissues, such as BAT, that show an intense oxidative metabolism. As already discussed, a highly active OXPHOS also leads to ROS formation. Notably, genetic and pharmacological experimental procedures that increase adipocyte ROS levels, or the related oxidation of cellular thiols, have been shown to stimulate adipocyte thermogenesis; in other words, a shift in the oxidative/reducing equilibrium is determined in BAT by induction of acute thermogenic respiration. It is worth noting that these modifications are reversible and do not cause a generalized oxidative stress [319]. Moreover, it seems that UCP1 is not the only actor in the mitochondrial proton leak and thermogenesis.

THs are essential for the thermogenic function of BAT, in synergy with the SNS [209,320]. BAT contains both TRα and TRβ, which seem to mediate the T3-dependent effects in synergy with the SNS and transcription of the UCP1 gene, respectively [209]. TRβ also stimulates UCP1 transcription in the WAT. In addition, in BAT, TH-dependent thermogenesis is stimulated by noradrenalin. This latter effect is due to stimulation of DIO2 through DIO2 gene transcription, on one hand, and stabilization of the protein, through its de-ubiquitination, on the other hand [209,321]. 

Like T3, 3,5-T2 also has an effect on BAT thermogenesis: (1) It improves survival in the cold of hypothyroid rats; (2) by directly binding, as already mentioned, to COX, it increases the oxidative potential of the cell; and (3) it induces an increase in mitochondrial biogenesis. Somehow, 3,5-T2 also has an effect on the number of noradrenergic fibers, and adrenergic stimulation is probably responsible for the 3,5-T2-dependent higher vascularization of BAT [209,322].

Most interesting, it has been shown that 3,5-T2 can induce “browning” in the WAT of T2-treated rats. With the term “browning” is described the fact that, when stimulated by physiological factors, such as THs, exercise, or diet, able to increase thermogenesis, WAT can acquire a BAT-like phenotype [323,324]. The process mainly occurs in the subcutaneous WAT and has been reported to involve some microRNAs (e.g., miR-133a and MiR196a) [324]. Recently, the effect of 3,5-T2 has been also studied, by proteomic analysis, in the visceral adipose tissue of HFD-rats, where a clear pro-angiogenic action was observed, together with reduction of proteins involved in lipid storage or in oxidative stress [325]. 

Although the mechanisms underlying browning have not been yet clarified, the process is of the utmost importance because an increase of BAT-like tissue also increases thermogenesis and, hence, stimulates the metabolism of the entire organism. Thus, to find factors able to induce browning could be an important way to improve metabolic disorders, such as metabolic syndrome and diabetes. In this context, 3,5-T2 seems to be a promising molecule.

### 4.6. The Ins and Outs of 3,5-T2 Research

In summary, a variety of experiments on model animals have clearly shown that 3,5-T2 behaves as a hormone. The peculiar property of 3,5-T2 is its mainly nucleus-independent activity. Although it can bind TRs, even if with a much lower affinity than T3, 3,5-T2′s main mechanism of action seems indeed to be based on a direct interaction with mitochondria, at the level of which 3,5-T2 is able to potentiate mitochondrial activity and thermogenesis. 

As discussed above, the treatment with 3,5-T2 may prevent the damage caused by HFD and reduce the circulating levels of LDL and TAG, as well as hepatic steatosis in overweight patients. It has been reported that the hormone may also reduce a pre-existing accumulation of fat in the rat liver [191]. In humans, a few studies have confirmed that the administration of 3,5-T2 can increase the resting metabolic rate, also reducing adiposity and body weight without side effects [250]. Interestingly, through molecular fingerprinting experiments, a strong positive association has been found between the 3,5-T2 levels in the human serum and compounds related to coffee consumption [326]. Taken together, all these finding are encouraging and provide evidence that 3,5-T2 might become a therapeutic agent of utmost importance. 

On the other hand, we still lack clear demonstration that the effects observed in mice/rats can be completely reproduced in our species. Experiments in humans are indeed still limited, and, as discussed, it is not yet clear whether 3,5-T2 is produced in peripheral tissues at significant concentrations It is not completely understood, for example, whether blood concentrations of 3,5-T2 are related or not to intracellular concentrations of the hormone: It is possible that T3 deiodination to 3,5-T2 is controlled by tissue factors, differently regulated, for example, in a tissue-specific and/or in a metabolic state-specific manner. From this point of view, it is intriguing that no significant difference was found in serum 3,5-T2 of hypothyroid and hyperthyroid patients [202] and that no significant correlation was evidenced between serum 3,5-T2 and the main THs [203]. As mentioned, a further problem arises because of contamination by T3 of the commercial preparations of 3,5-T2, used as standards in the measurements [20,204]. 

Even with these limitations, 3,5-T2 is widely considered a bioactive molecule [20,59,113,191,204,205,206,207,208,209,210,211]. Thus, in summary, the field is very promising and might offer important advancements in the therapy of obesity and related pathologies. However, it is mandatory to conclusively understand how intracellular 3,5-T2 is produced and how this production is regulated. Moreover, as 3,5-T2 can be transformed into other metabolites, the entire metabolic process in which this hormone is involved should be clarified. 

## 5. Conclusions and Perspectives

**Figure 7:**
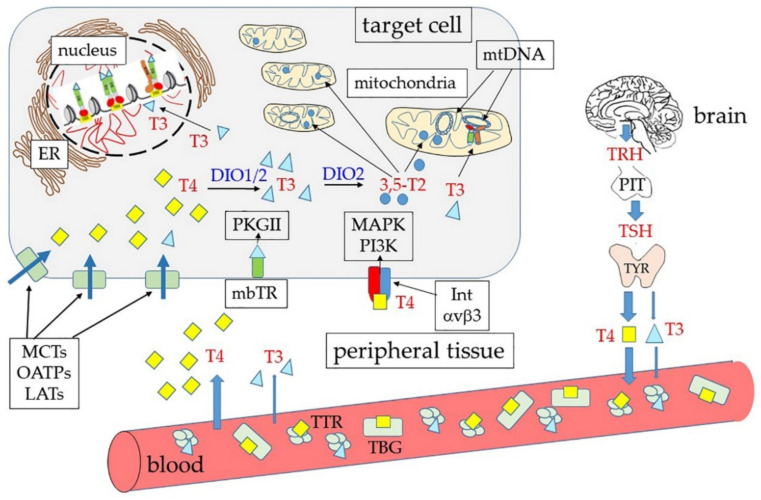
General summary of T4 (yellow squares), and T3 (light blue triangles) production and transport to the peripheral tissues. As indicated by the thickness of the light blue arrows, T4 is released from the gland, and circulates in the blood at a much higher concentration than T3. In the blood, two main TH transporters are present: the thyroxine-binding globulin (TBG), and transthyretin (TTR). THs enter the cells through transmembrane transporter proteins, such as the monocarboxylate transporters (MCTs), the organic anion transporters (OATPs), and the L-type amino acid transporters (LATs). Once in the target cell, T4 is deiodinated by deiodinases 1 and 2 (DIO1/2) to T3. T3 is the most active form since it binds with the highest affinity to the nuclear receptors. In addition, T3 probably also binds some truncated forms of receptors targeted to mitochondria, where T3 might regulate also transcription of genes present in the mitochondrial DNA (mtDNA). T3 can be also deiodinated to 3,5-T2 and this latter molecule can reach the mitochondria that seem to represent its main target. Notably, THs (and especially T4, given its much higher concentration in the circulation) can also bind integrin αvβ3 (Int αvβ3), thus activating an intracellular pathway that involves the Mitogen-activated protein kinase (MAPK), and the PhosphatidylInositol-3-Kinase (PI3K). Moreover, truncated forms of TRs are palmitoylated and inserted into the inner leaflet of the plasma membrane (membrane-bound TRs, mbTR), from which they can bind THs and activate a pathway that involves the cGMP-dependent protein kinase II (PKGII) (TR).

As discussed above, in the last two decades, a multitude of studies have highlighted that the effects of THs on cells do not rely only on their nuclear action, but also on a variety of other pathways, sometimes involving their metabolites. Among these latter molecules, 3,5-T2 has attracted much interest since it was found to be effective in increasing the resting energy. Most important, 3,5-T2 seems to have a very rapid effect, probably due to its ability to interact directly with mitochondria. In addition, at least at low concentration, it does not seem to cause thyrotoxic effects [20,188]. A general summary of TH metabolism and effects in the target cell, including formation and mitochondrial targeting of 3,5-T2, is shown in Figure 7. 

Notably, beside 3,5-T2, other TH endogenous metabolites have been reported to be active and to have interesting effects on a variety of cellular activities. For example, 3,5,3′-triodothyroacetic acid (TRIAC), derived from deamination of T3, can bind both TRβ and TRα with similar or even higher affinity than T3 and has been proposed as an alternative TR ligand for therapeutic aims, also because it does not use MCT8 as a membrane transporter, and can thus offer an opportunity to treat the Allan–Herndon–Dudley syndrome, a developmental disorder that affects the central nervous system and is due to an MCT8 mutation [20,113]. Similarly, 3,3’,5,5’-tetraiodothyroacetic acid (TETRAC), derived from deamination of T4, is a powerful ligand of αvβ3 receptor, acting as an efficient competitor for T4/T3 binding, thus inhibiting their effects in a variety of cellular models of tumorigenesis [20,327]. Even rT3 has been reported to induce non-genomic responses in Sertoli cells [328]. Finally, THs can also undergo decarboxylation, giving rise to thyronamines, such as 3-iodothyronamine (T1AM). Recently, it was reported that decarboxylation is catalyzed by ornithine decarboxylase (ODC) that seems to use as substrates only T4 and 3,5-T2 [329]. In detail, the biosynthetic pathway should involve TH deiodination to 3,5-T2, followed by its decarboxylation and further deiodination by DIO3 to T1AM [210]. Thus, 3,5-T2 should be the T1AM’s immediate precursor. Although the membrane transporters for T1AM have not been yet identified, it has been suggested that, given its ability to bind low-density lipoproteins (LDL), its cellular uptake might be coupled to LDL endocytosis [210,330]. T1AM does not bind TRs, but is a high-affinity agonist of trace amine-associated receptor 1 (TAAR1), a membrane G protein-coupled receptor, and probably also of other members of the family. As it happens for other aromatic amines (cathecolamines, serotonine, etc.), T1AM seems to be a signal molecule with a variety of effects, such as: (1) Reduced heart rate and contractility. (2) decreased insulin secretion and increased glucagon secretion, (3) lipid catabolism, (4) pro-learning effects, and (5) decreased proliferation of tumor cell lines [210,331]. 

Notably, the fact that T1AM derives from 3,5-T2 suggests that some of the effects of this latter molecule might be mediated by T1AM.

In conclusion, it is now clear that the canonical mechanisms of action of THs are no more sufficient to explain all the effects of these important hormones and that at least some of their metabolites are also involved in their activities. If, on one hand, these discoveries opened an incredibly wide field of analysis, at the same time, we are now realizing that these novel thyroid hormones can offer unforeseen opportunities in the therapy of a variety of human metabolic disorders, provided that we can clearly understand how they are produced, how they are catabolized, and how their action is modulated in our body.
